# Subpopulations of miniature pig mesenchymal stromal cells with different differentiation potentials differ in the expression of octamer-binding transcription factor 4 and sex determining region Y-box 2

**DOI:** 10.5713/ajas.19.0416

**Published:** 2019-10-21

**Authors:** Ryounghoon Jeon, Sungjo Park, Sung-Lim Lee, Gyu-Jin Rho

**Affiliations:** 1 Department of Theriogenology and Biotechnology, College of Veterinary Medicine, Gyeongsang National University, Jinju 52828, Korea; 2 Department of Cardiovascular Medicine, Mayo Clinic, Rochester, MN 55905, USA

**Keywords:** Miniature Pig, Mesenchymal Stromal Cell (MSC) Subpopulations, Octamer-binding Transcription Factor 4 (*OCT4*), Sex Determining Region Y-box 2 (*SOX2*), Aging

## Abstract

**Objective:**

Human mesenchymal stromal cells (MSCs) exhibit variable differentiation potential and can be divided accordingly into distinct subpopulations whose ratios vary with donor age. However, it is unknown whether the same is true in pigs. This study investigated MSC subpopulations in miniature pig and compared their characteristics in young (2 to 3 months) and adult (27 to 35 months) pigs.

**Methods:**

Osteogenic, chondrogenic, and adipogenic capacity of isolated MSCs was evaluated by von Kossa, Alcian blue, and oil red O staining, respectively. Cell surface antigen expression was determined by flow cytometry. Proliferative capacity was assessed with the 3-(4,5-dimethylthiazol-2-yl)-2,5-diphenyltetrazolium bromide assay. Expression of marker genes was detected by quantitative real-time polymerase chain reaction.

**Results:**

Porcine MSCs comprised cells with trilineage and bilineage differentiation potential (tMSCs and bMSCs, respectively) and non-differentiating stromal cells (NDSCs). The tMSC and bMSC fractions were smaller in adult than in young pigs (63.0% vs 71.2% and 11.6% vs 24.0%, respectively, p<0.05); NDSCs showed the opposite trend (25.4% vs 4.8%; p<0.05). Subpopulations showed no differences in morphology, cell surface antigen expression, or proliferative capacity, but octamer-binding transcription factor 4 (*OCT4*) expression was higher in tMSCs than in bMSCs and NDSCs (p<0.05), whereas sex determining region Y-box 2 (*SOX2*) expression was higher in tMSCs and bMSCs than in NDSCs (p<0.05). Aging had no effect on these trends.

**Conclusion:**

Porcine MSCs comprise distinct subpopulations that differ in their differentiation potential and OCT4 and SOX2 expression. Aging does not affect the characteristics of each subpopulation but alters their ratios.

## INTRODUCTION

Since the first isolation from bone marrow, mesenchymal stem cells (MSCs) have been characterized for their stemness characteristics mainly focused on their plastic adherence, tri-lineage differentiation potential as well as expression for the cluster of differentiation (CD) markers [1]. The MSCs can be used for tissue repair in regenerative medicine owing to their ability to differentiate into mesenchymal lineages such as bone, cartilage, and tendon, regulate immune responses in areas of damaged tissue by inhibiting tumor necrosis factor expression, interleukin-10 production, and T cell activity, and create an anti-apoptotic, angiogenic, and mitogenic microenvironment [2].

The MSCs can be isolated based on their ability to adhere to a plastic dish. However, various types of stromal cells also have this property making it challenging to isolate pure MSC cultures [3,4]. Consequently, MSCs isolated in this manner exhibit variable characteristics depending on the original progenitor cells [5]. Using MSCs without considering this heterogeneity can decrease their treatment efficiency and reproducibility since the proportion of effective cells will be reduced and the cell population will include uncharacterized cells, which can lead to safety issues such as increased embolism due to the use of more cells than necessary to achieve the desired effects [6]. Many studies have been carried out to characterize the heterogeneity of MSCs in terms of differentiation potential, molecular state, and biophysical properties [7,8]. Although it was originally thought that all MSCs exhibit osteogenic, chondrogenic, and adipogenic capacities, it was later shown that these cells comprise numerous subpopulations with distinct cell fate programs [1,7]. However, other than their capacity to differentiate into specific lineages, no studies have fully characterized the features of these subpopulations. Identifying molecular markers for each subpopulation is important to increase the purity and uniformity of MSC pools [9].

The age of MSC donors is difficult to control, especially due to the requirement of using autologous cells to prevent immune reactions [10]. Donor age has been shown to affect MSC properties [7,11], and MSC differentiation potential was reported to decrease with donor age, although most of these studies have not considered the heterogeneity of MSCs [10,12, 13], which is critical for their successful clinical application [9,13].

Since pigs are anatomically and physiologically similar to humans, they are an ideal model organism for biomedical research and have been used for pathological studies, pharmacological assessment, and xenotransplantation studies, as well as for preclinical studies in regenerative medicine [14]. Miniature pigs are smaller than regular domestic pigs and are thus easier to handle [15,16]. However, compared to human MSCs, there is relatively little known about the heterogeneity of porcine MSCs.

To this end, the present study investigated the character istics of miniature pig MSC subpopulations, and the effect of donor age on their properties. MSCs were isolated from young and adult miniature pigs and cultured as individual colonies that were classified into three distinct subpopulations according to their osteogenic, chondrogenic, and adipogenic capacity. These subpopulations were compared in terms of MSC-specific surface marker and pluripotency marker expression and proliferative capacity.

## MATERIALS AND METHODS

### Materials and ethics approval

All chemicals and media were purchased from Thermo Fisher Scientific (Waltham, MA, USA) unless specified otherwise. Experiments were approved by the research ethics committee of Gyeongsang National University Animal Center for Biomedical Experimentation (GNU-130308-P0022).

### Isolation and *in vitro* culture of MSCs

Bone marrow extracts were obtained from young (2 to 3 months) and adult (27 to 35 months) male miniature pigs (n = 5 each; PWG Genetics, Seoul, Korea). MSCs were isolated as individual colonies according to a previously described protocol [17,18], with some modifications. A 2-mL volume of bone marrow aspirate was diluted with 2 mL of Dulbecco’s phosphate-buffered saline, placed on a Ficoll (Ficoll-Paque PLUS; GE Healthcare, Little Chalfont, UK) layer, and centrifuged (400×g, 30 min, 4°C). The interphase layer containing mononuclear cells was collected and treated with ammonium chloride (160 mM) to lyse erythrocytes. Mononuclear cells were resuspended in complete medium composed of Dulbecco’s modified eagle’s medium supplemented with 1% GlutaMax, 100 U/mL penicillin, 100 μg/mL streptomycin, and 10% fetal bovine serum, plated onto 100-mm tissue culture dishes, and incubated at 38°C with 5% CO_2_. After 24 h, non-adherent cells were removed by changing the medium, and individual colonies of fibroblast-like cells were isolated using cylinders with silicone grease (Corning Inc., Corning, NY, USA). Cells were passaged at a 1:4 ratio by trypsin digestion when they reached 70% to 80% confluence. All experiments were performed using passage 3 to 5 cells.

### *In vitro* differentiation

To evaluate differentiation potential, MSCs were induced to form osteoblasts, chondroblasts, and adipocytes for 3 weeks using previously described protocols [18]. Osteogenesis medium was comprised of 0.1 μM dexamethasone, 0.2 mM ascorbic acid 2-phosphate, and 10 mM glycerol 2-phosphate, and mineralization was confirmed by von Kossa staining. Adipogenesis cells were induced with culture medium containing 1 μM dexamethasone, 10 μM insulin, and 100 μM indomethacin, and intracellular lipid droplet formation was confirmed by oil red O staining. To induce chondrogenesis, 1×10^5^ cells were resuspended in StemPro chondrocyte differentiation medium, transferred to 15-mL conical tubes, and centrifuged (400×*g*, 4 min); extracellular proteoglycan in sections of the pellets was detected by 1% Alcian blue staining.

### Immunophenotyping

Cells were analyzed for the expression of mesenchymal markers (CD73, CD90, and CD105) and hematopoietic markers (CD34 and CD45) as described previously [1,18]. The MSCs at 70% to 80% confluence were harvested and fixed with 3.7% paraformaldehyde for 30 min at room temperature, then incubated with fluorescein isothiocyanate (FITC)-conjugated mouse anti-CD34, anti-CD45, anti-CD73, anti-CD90, anti-CD105, and isotype control antibodies (diluted 1:200; BD Pharmingen, CA, San Jose, CA, USA) for 1 h at room temperature. FITC-labeled cells (1×10^4^) were analyzed by flow cytometry on a FACS Calibur instrument (BD Biosciences, Franklin Lakes, NJ, USA) using Cell Quest Pro v.3.3 software (BD Biosciences, USA).

### Proliferative capacity

Cell proliferation was quantified with the 3-(4,5-dimethylthiazol-2-yl)-2,5-diphenyltetrazolium bromide (MTT) cell proliferation assay kit (Molecular Probes, Ashland, OR, USA) according to the manufacturer’s instructions. Cells (1×10^3^/well) were seeded in 96-well plates in 100 μL of complete culture medium, and cell proliferation was evaluated 1, 3, 5, 7, 9, 11, 13, and 15 days later. A 10-μL volume of 12 mM MTT solution was added to each well and the plate was incubated at 38°C for 4 h; the culture medium was then removed and 50 μL of dimethyl sulfoxide was added, which was followed by incubation for 10 min. The absorbance of dissolved MTT metabolite was measured at 540 nm with a spectrophotometer (FLUOstar Omega; BMG Labtech, Ortenberg, Germany).

### Gene expression profiling

The expression levels of the following genes were analyzed by quantitative real-time–polymerase chain reaction (qRT-PCR): pluripotency genes including, octamer-binding transcription factor 4 (*OCT4*), sex determining region Y-box 2 (*SOX2*), and Nanog homeobox (*NANOG*); osteocyte-specific genes runt-related transcription factor 2 (*RUNX2*) and osteonectin (*ON*); chondrocyte-specific genes aggrecan (*ACAN*) and collagen type II α1 (*COL2A1*) and adipocyte-specific genes peroxisome proliferator-activated receptor γ (*PPARG*) and fatty acid-binding protein 4 (*FABP4*). Primer sequences are listed in Table 1. Cultured cells were harvested and stored at −80°C until further use. Total RNA was isolated using the RNeasy Mini Kit (Qiagen, Hilden, Germany) and quantified with a Nanodrop-1000 spectrophotometer (Thermo Fisher Scientific, USA). The RNA (1 μg) was reverse-transcribed into cDNA using the SuperScript IV First-Strand Synthesis System (Thermo Fisher Scientific, USA) according to the manufacturer’s instructions, and qRT-PCR was performed on a Viia 7 real time-PCR instrument (Thermo Fisher Scientific, USA) with RT^2^ SYBR Green ROX qPCR Mastermix (Qiagen, Germany), 0.5 μM forward and reverse primers, and 0.1 μg of cDNA per reaction. The reaction conditions were as follows: 95°C for 10 min; 40 cycles at 95°C for 15 s and 60°C for 60 s; 60°C to 95°C at 1°C/s; 40°C for 30 s. The hydroxymethylbilane synthase gene was used for relative quantification of transcript levels and Ct values were analyzed using QuantStudio software (Applied Biosystems, Carlsbad, CA, USA).

### Statistical analysis

Data are expressed as mean±standard deviation. Differences between two groups were evaluated with the Student’s t test and differences among multiple groups were evaluated by one-way analysis of variance followed by Tukey’s post-hoc test using Prism 7 software (GraphPad Inc., La Jolla, CA, USA). Statistical significance was set at p<0.05.

## RESULTS

### Mesenchymal stromal cell isolation and expansion

**Figure 1:**
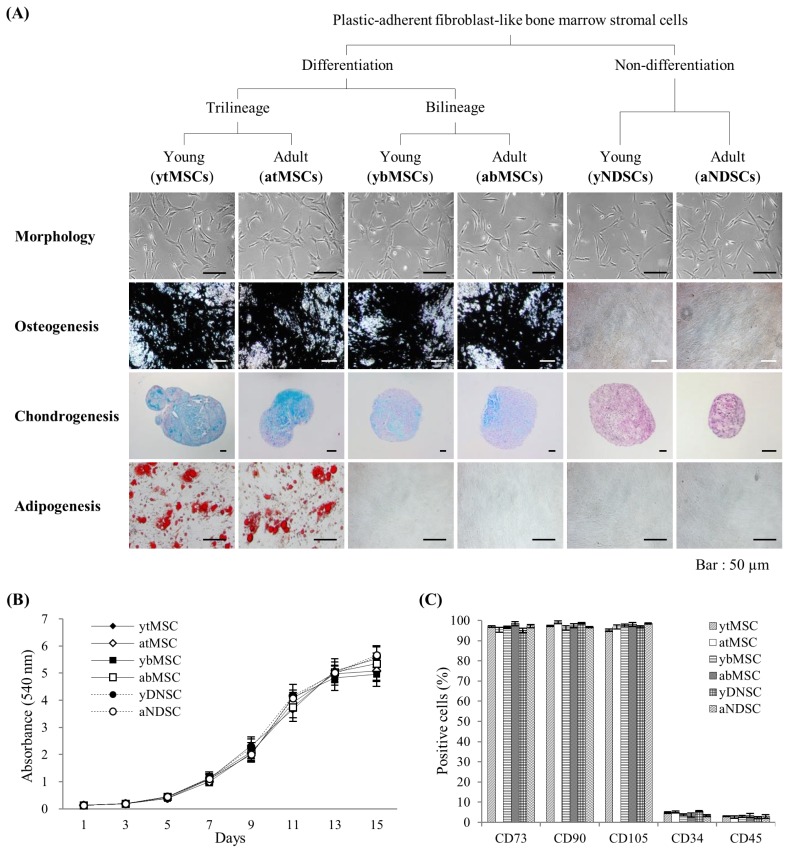
Hierarchy and phenotype of mesenchymal stromal cell (MSC) subpopulations. (A) MSC subpopulations were categorized according to mesenchymal lineage differentiation potential and age. The morphology of cultured cells was examined by phase-contrast microscopy at 24 h after plating. Osteogenesis, chondrogenesis, and adipogenesis were induced for 3 weeks and confirmed by von Kossa, Alcian blue, and oil red O staining, respectively. Scale bar = 50 μm. (B) *In vitro* growth kinetics of MSC subpopulations. Cell proliferation was evaluated at 1, 3, 5, 7, 9, 11, 13, and 15 days after plating with the MTT assay by measuring the absorbance at 540 nm. (C) Surface marker expression was analyzed by flow cytometry. CD73, CD90, and CD105 served as MSC-positive markers and CD34 and CD45 served as MSC-negative markers. Data are shown as mean %±standard deviation of five replicates. CD, cluster of differentiation; MTT, 3-(4,5-dimethylthiazol-2-yl)-2,5-diphenyltetrazolium bromide.

The MSCs isolated from all the experimental groups were evaluated for their stemness characteristics as per the guidelines given by Dominici et al [1] Using similar approach, isolated MSCs were first evaluated for their plastic adherence and morphology. Experiments were performed to evaluate the characteristics of individual colonies of MSCs isolated from bone marrow. Fibroblast-like cells that adhered to the culture dish were observed starting from day 3. On day 7, a total of 91 and 83 individual colonies were obtained from young and adult pig bone marrow samples, respectively. The mean number of colonies isolated from each individual was 18.2±5.7 and 16.6±4.5 from young and adult animals, respectively, with no age-related differences. All adherent cells had a similar fibroblast-like morphology (Figure 1A).

### Differentiation characteristics of isolated mesenchymal stromal cells

To investigate the lineage-specific differentiation potential of the isolated cells, osteogenic, chondrogenic, and adipogenic differentiation was induced in 174 individual clones isolated from young and adult bone marrow samples (Table 2) and the identity of each lineage was confirmed by von Kossa, Alcian blue, and oil red O staining, respectively (Figure 1A). The clones were classified as MSCs with trilineage differentiation potential (tMSCs), MSCs with osteo-chondrogenic bilineage differentiation potential (bMSCs), and non-differentiating stromal cells (NDSCs). Osteo-adipogenic, chondro-adipogenic, and pure osteogenic, chondrogenic, and adipogenic phenotypes were not observed in any of the examined clones (Table 2). The identified subpopulations were also analyzed for cell surface and pluripotency marker expression and proliferative capacity. Aging reduced the size of the tMSC (p<0.05) and bMSC (p<0.01) populations and increased that of the NDSC population (p<0.01).

### Proliferative capacity of isolated mesenchymal stromal cells

Proliferative capacity is not only a criterion for identifying MSCs but has clinical importance since it will determine the total number of available cells. The MTT assays were performed to compare the proliferative capacity of the different MSC subpopulations, as well as the effect of aging on this property. The growth kinetics of the three MSC subpopulations did not differ according to differentiation potential or age (Figure 1B).

### Cell surface antigen expression in isolated mesenchymal stromal cells

The MSCs are defined by expression of the cell surface antigens CD73, CD90, and CD105 and the absence of CD34 and CD45 expression. The tMSC clones satisfying the differentiation criteria of MSCs showed the appropriate MSC antigen profile. However, regardless of differentiation potential or donor age, all three MSC subpopulations expressed the expected markers (Figure 1C). Thus, the surface antigen profile used to define MSCs did not distinguish subpopulations with distinct differentiation potentials.

### Gene expression profiling of isolated mesenchymal stromal cells

**Figure 2:**
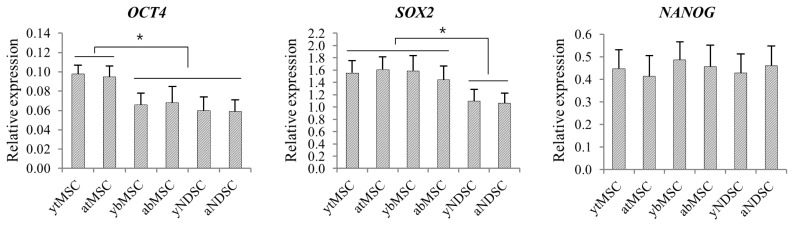
Transcript expression of pluripotency-related transcription factors in mesenchymal stromal cells. Transcript levels were analyzed by qRT-PCR and normalized to that of *HMBS* transcripts. Data are shown as mean %±standard deviation of five replicates. * p<0.05. qRT-PCR, quantitative real-time polymerase chain reaction; *HMBS*, hydroxymethylbilane synthase.

The expression of the pluripotency genes *OCT4*, *SOX2*, and *NANOG* in MSCs was then investigated (Figure 2). *OCT4* expression was higher in tMSCs than in the other subpopulations (p<0.05), whereas no difference was observed between bMSCs and NDSCs. *SOX2* expression was higher in tMSCs and bMSCs than in NDSCs (p<0.05), with no difference between the former two MSC types. There was no difference in *NANOG* level among groups. MSC subpopulations did not show any age-dependent differences in the expression of pluripotency genes.

**Figure 3:**
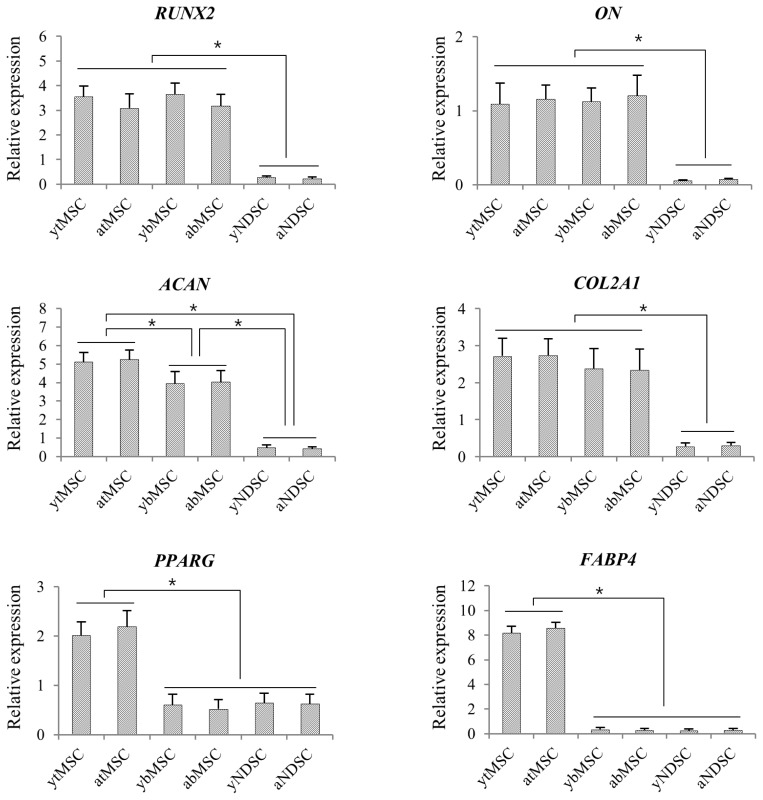
Transcript expression of lineage-specific genes during induction in mesenchymal stromal cells. Transcript levels were analyzed by qRT-PCR 3 weeks after inducing osteoblast, chondroblast, and adipocyte differentiation, and normalized relative to that of *HMBS*. Data are shown as mean %±standard deviation of five replicates. * p<0.05. qRT-PCR, quantitative real-time polymerase chain reaction; *HMBS*, hydroxymethylbilane synthase.

Lineage-specific gene expression was investigated after 3 weeks of differentiation by quantifying relative transcript levels of genes related to osteogenesis, chondrogenesis, and adipogenesis. The results were in accordance with those obtained by histological analysis (Figure 3). The osteogenesis-related genes *RUNX2* and *ON* were expressed at higher levels in tMSCs and bMSCs than in NDSCs (p<0.05); moreover, *RUNX2* levels were higher in young than in adult tMSCs and bMSCs, although the difference was not statistically significant. For the chondrogenesis-related gene *ACAN*, the rank order of expression level was tMSC>bMSC>NDSC (p<0.05). In contrast, *COL2A1* expression was similar in tMSCs and bMSCs, and was higher in these two subpopulations than in NDSCs (p<0.05). The adipogenesis-related genes *PPARG* and *FABP4* were more highly expressed in tMSCs than in bMSCs and NDSCs, with no difference in levels between the latter two (p<0.05). The trend observed in each subpopulation was the same irrespective of whether the cells were derived from young or adult donors.

The results of the present study revealed that both the young and adult animals derived MSC subpopulations shared similar morphological, cell surface profiling and growth characteristics irrespective of their donor age. However, tMSCs showed high expression levels of *OCT4* and *SOX2* pluripotency markers than NDSCs. Like tMSCs, bMSCs showed comparable expression. Similarly, tMSCs followed by bMSCs were evaluated with high expression levels of linear specific markers. Therefore, subpopulation of MSCs with high potency (tMSCs and bMSCs) could be preferred to get highly efficient and desirable results. However, present study lacks the further evaluation of isolated cell subpopulations for their efficiency for other specific lineages such as neurons, hepatocytes, muscles etc. for which further elucidation will be needed.

## DISCUSSION

In the present study, subpopulation of MSCs isolated from young and adult miniature pigs were classified based on their differentiation potential as tMSCs, bMSCs, and NDSCs, which could also be distinguished by their differential expression of *OCT4* and *SOX2* before and lineage-specific genes after differentiation. In contrast, the subpopulations showed comparable cell morphology, proliferative capacity, and surface antigen expression. Although young and adult cells differed in terms of the proportion of colonies in each subpopulation, there were no age-related differences in MSC characteristics. These findings indicate that *OCT4* and *SOX2* can serve as molecular markers for the qualitative assessment of MSC subpopulations and that aging alters their ratios but not MSC characteristics.

As previously reported, in this study, the number of colo nies was independent of donor age and individual colonies could not be distinguished by cell morphology [10,19]. Thus, the heterogeneity of MSCs cannot be detected during isolation and it is necessary to evaluate other MSC characteristics. The proportion of tMSCs and bMSCs decreased with increasing donor age, whereas the fraction of NDSCs increased by >5-fold. This agrees with the findings of a previous report on the proportions of human MSC subpopulations and the effects of aging on these ratios [7,9]. Results of this study suggest that the age-dependent changes in MSC differentiation potential are due to these alterations in subpopulation ratios [7,10,11,13]. To address the discrepancies in previous studies regarding whether MSC differentiation potential decreases or is maintained with donor aging, it is necessary to consider the proportions of MSC subpopulations used in each study [7,9]. In accordance with the staining results for each subpopulation, the post-differentiation expression of lineage-specific genes was higher. Interestingly, chondrogenesis-specific genes were expressed at lower levels in young and adult bMSCs than in young and adult tMSCs. This suggests that the specification of chondrogenic fate in MSCs is not independent of the specification of other lineages, as suggested by a previous report [20]. In contrast, *RUNX2* expression in both tMSCs and bMSCs decreased with aging, albeit without statistical significance. Additional research is needed to clarify the molecular basis for changes in differentiation potential with respect to MSC donor aging.

Proliferation is a key stem cell characteristic that is critical for the repair of damaged tissues and maintenance of stem cell populations [21]; it is also a useful feature for increasing the fraction of specific cell types through serial passaging [22]. However, divergent findings regarding the proliferative capacity of aged MSCs have been reported, and the relationship between proliferative capacity and differentiation potential among MSC subpopulations is unclear [9,11]. In this study, no differences were found in the proliferative capacities of the different subpopulations regardless of differentiation potential and donor age. Therefore, this property cannot be used to distinguish between specific MSC subpopulations. There are two possible explanations for such discrepant findings: first, unlike previous studies that used the limited dilution or other methods, the present study isolated individual colonies, which eliminated the influence of other colonies in the same culture and preserved the original characteristics of that colony [7,9,17]. Second, MSCs from pigs that were on average 30.6 months of age might not have reached cellular senescence, and therefore older pigs should be examined in future studies.

Positive expression of MSC specific CD markers while lacking the hematopoietic cell markers is a predefined criterion [1]. In this study, surface marker profiles were similar among the different MSC subpopulations regardless of donor age and the differentiation potential. Various markers including STRO1, CD106, SSEA4, CD56, CD271, MSC antigen 1, D7FIB, and CD146 are also used to complement the typical MSC markers and characterize the functional properties of MSCs, and can distinguish MSCs from cells of other lineages such as hematopoietic stem cells; however, these markers lack the specificity and selectivity to discriminate among MSC subpopulations with distinct differentiation potentials [4,6]. Therefore, to isolate homogenous MSCs or to investigate MSC subpopulation characteristics, additional markers are needed that could reflect the differentiation potential of a specific MSC subpopulation.

Pluripotency is the capacity to differentiate into all cell types in the body and this decreases with differentiation and is therefore a useful feature to identify stem cell populations [18,23]. Elevated expression of pluripotency genes including *OCT4*, *SOX2*, and *NANOG* is a characteristic of embryonic stem cells and induced pluripotent stem cells [23,24], but in the case of MSCs, the expression and function of these genes remains controversial and they are therefore not used as a criterion for MSC identification [1,25]. Nevertheless, since pluripotency genes have a major influence on differentiation and proliferation, analyzing their expression can broaden the knowledge of cellular phenotypes and the mechanisms of aging [18,26]. *OCT4* is a master transcriptional regulator that suppresses the differentiation of pluripotent stem cells and maintains their stemness; it plays a similar role in MSCs and is associated with cell cycle regulation and cell plasticity [18,20,27]. In the present study, *OCT4* expression was increased only in tMSCs, which have trilineage differentiation potential; this supports the previous finding that *OCT4* overexpression enhances adipogenesis [28]. Since *OCT4* levels did not differ between bMSCs and NDSCs, high OCT4 expression might be useful for identifying MSC subpopulations with the potential to undergo osteogenic, chondrogenic, and adipogenic differentiation. The expression of *SOX2*, along with that of *OCT4* was downregulated in senescent MSCs with reduced differentiation potential, indicating that *SOX2* plays a critical role in differentiation [18]. Furthermore, *SOX2* is known to participate in cell proliferation by regulating the expression of c-MYC [20]. In this study, *SOX2* expression did not differ among MSC subpopulations. However, since it was higher in MSCs with the ability to differentiate into two or three lineages than in NDSCs, it could serve as a marker for MSC subpopulations with osteogenic and chondrogenic differentiation potential [29]. However, in contrast to a previous report that *SOX2* is involved in both differentiation and proliferation, a clear correspondence between *SOX2* levels and proliferative capacity was not observed, possibly because the adult pigs used as an aging model in this study showed senescence-associated changes in gene expression but not proliferative capacity [20,30]. *NANOG* was previously detected only in MSCs proliferating in culture [26], and its expression suppressed the increase in doubling time and delayed senescence in adult MSCs [27,30]. In this study, no difference was found in *NANOG* expression in pig MSCs despite differences in differentiation potential, in accordance with the lack of a difference in proliferative capacity. Thus, the expression of *OCT4* and *SOX2* but not of *NANOG* might be useful to classify bone marrow MSC subpopulations.

This is the first report characterizing MSC subpopula tions derived from miniature pigs. These results can be used to improve purification strategies of MSC subpopulations and increase the efficiency of MSC usage from aged donors. However, since this study was carried out under *in vitro* conditions, the origin and basis for MSC heterogeneity *in vivo* remain unclear, and future studies should identify and standardize markers for specific MSC subpopulations to improve their clinical application.

## Figures and Tables

**Table 1:** Primer sequence for gene expression analysis

Target gene	Sequence 5′-3′	Amplicon size (bp)	Reference
*OCT4*	F-AGTCCCAGGACATCAAAGCG	129	NM_001113060.1
	R-CCTCCCAAAGAGAACCCCC		
*SOX2*	F-AGGACCAGCTGGGCTATCCG	170	NM_001123197.1
	R-GCCCTGCTGCGAGTAGGACA		
*NANOG*	F-AACCAAACCTGGAACAGCCAGAC	152	NM_001129971.1
	R-GTTTCCAAGACGGCCTCCAAAT		
*RUNX2*	F-CATCCATCCACTCCACCACC	134	XM_005666074.3
	R-ACTGAGAGTGGAAGGCCAGA		
*ON*	F-ACAGACGCTCTCGCCTAAAC	185	NM_001031794.1
	R-ACCCCTGTCGGATGTAGTGA		
*ACAN*	F-GAAGGTTGCTACGGGGACAA	113	NM_001164652.1
	R-ACCTCACCCTCCATCTCCTC		
*COL2A1*	F-ACCTGAAAGACTGCCTCAGC	103	XM_021092611.1
	R-TGTCCCTTTGGTCCCAGTTG		
*PPARG*	F-CTGTGGACCTGTCGGTGATG	123	NM_214379.1
	R-GATCAGCTCTCGGGAATGGG		
*FABP4*	F-GAAAGGTGTCACGGCTACCA	103	NM_001002817.1
	R-TGTCGGGACAATACATCCAACA		
*HMBS*	F-CGCAACGGCGGAAGAAAATA	113	NM_001097412.1
	R-ATAAGGCTTTCAGCGTTGCC		

*OCT4*, octamer-binding transcription factor 4; *SOX2*, sex determining region Y-box 2; *NANOG*, NANOG homeobox; *RUNX2*, runt-related transcription factor 2; *ON*, osteonectin; *ACAN*, aggrecan; *COL2A1*, collagen type II alpha 1; *PPARG*, peroxisome proliferator-activated receptor gamma; *FABP4*, fatty acid-binding protein 4; *HMBS*, hydroxymethylbilane synthase.

**Table 2:** Classification and differentiation potential of bone marrow-derived adherent cells

Differentiation			Number of clones (mean %±SD)	
	
Osteogenesis	Chondrogenesis	Adipogenesis	Young	Adult
+	+	+	64 (71.2±4.5)	53 (63.0±3.6)*
+	+	−	22 (24.0±3.7)	10 (11.6±2.8)**
+	−	+	0 (0)	0 (0)
−	+	+	0 (0)	0 (0)
+	−	−	0 (0)	0 (0)
−	+	−	0 (0)	0 (0)
−	−	+	0 (0)	0 (0)
−	−	−	5 (4.8±2.8)	20 (25.4±4.2)**
	Total		91	83

SD, standard deviation; +, Capacity to differentiate into the specific lineage; −, inability to differentiate into the specific lineage.

* p<0.05,

** p<0.01 vs young group.
